# From in vitro to in vivo: Integration of the virtual cell based assay with physiologically based kinetic modelling

**DOI:** 10.1016/j.tiv.2017.06.015

**Published:** 2017-12

**Authors:** Alicia Paini, Jose Vicente Sala Benito, Jos Bessems, Andrew P. Worth

**Affiliations:** Chemical Safety and Alternative Methods Unit, EURL ECVAM, Directorate F – Health, Consumers and Reference Materials, Joint Research Centre, European Commission, Ispra, Italy

**Keywords:** VCBA, PBK, PBD, Estragole, DNA adduct, Risk assessment, KNIME workflow

## Abstract

Physiologically based kinetic (PBK) models and the virtual cell based assay can be linked to form so called physiologically based dynamic (PBD) models. This study illustrates the development and application of a PBK model for prediction of estragole-induced DNA adduct formation and hepatotoxicity in humans. To address the hepatotoxicity, HepaRG cells were used as a surrogate for liver cells, with cell viability being used as the in vitro toxicological endpoint. Information on DNA adduct formation was taken from the literature. Since estragole induced cell damage is not directly caused by the parent compound, but by a reactive metabolite, information on the metabolic pathway was incorporated into the model. In addition, a user-friendly tool was developed by implementing the PBK/D model into a KNIME workflow. This workflow can be used to perform in vitro to in vivo extrapolation and forward as backward dosimetry in support of chemical risk assessment.

## Highlights

• VCBA is coupled with PBK model linking intracellular concentration to external dose.
• The integrated model is applied to perform IVIVE of estragole-induced cell damage.
• The IVIVE process is automated by means of a KNIME workflow.

## Graphical abstract

**Figure:**
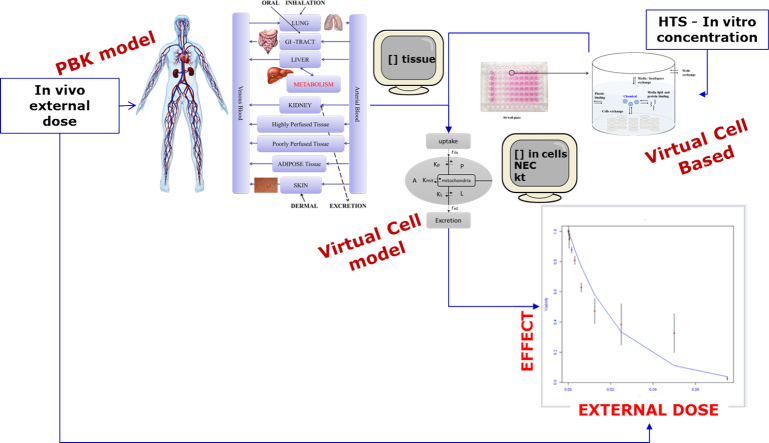

## 1 Introduction

The use of alternatives to animal testing is becoming increasingly important in the safety assessment of chemicals. Various cellular models have been developed, such as human cell lines (e.g. HepG2, HepaRG), freshly isolated hepatocytes, co-cultures, 3D models, and tissue/organ on chip models (Soldatow et al., 2013, Ramanan et al., 2014). In order to use human in vitro concentration-response data for human risk assessment, a stronger focus on internal exposure is necessary. In such an approach, in vitro concentrations (points of departure) would be compared to simulated or measured (e.g. by biomonitoring) human in vivo systemic exposure concentrations. The resulting margin of internal exposure (MOIE) would then be used to characterise the risk (Bessems et al., 2017, Bessems et al., 2015).

Computational approaches that aid the risk characterization are: i) Quantitative structure–activity relationship (QSAR) models, which relate the biological activity of a compound to its molecular structure; ii) physiologically based kinetic and dynamic (PBK/D) modelling, which can be used to simulate the biokinetics and dynamics of chemicals in model organisms or humans; and iii) other biokinetic mathematical models, such as the virtual cell based assay (VCBA), to simulate fate of chemical taking into account the setup of in vitro experiments, leading to intracellular simulation of the chemical under study.

Despite considerable progress in the development of these in silico approaches, there is still a need to develop practical tools for risk assessors, who increasingly need to rely on in vitro to in vivo extrapolations of chemical toxicity in order to carry out animal-free safety assessments. Recent work has illustrated several approaches to perform Quantitative In vitro to In vivo Extrapolation (QIVIVE) (Broeders et al., 2014). Uncertainties in the extrapolation from in vitro concentrations to in vivo human exposure conditions, including the influence of differences between species and exposure routes, can be taken into account by an integrated approach. Such an approach is to link the two models - the VCBA with the PBK model. Briefly, the VCBA model describes and predicts what is happening in an in vitro system, especially the fate of a chemical within the well, taking into account partitioning with protein, lipids, and plastic binding (Zaldívar Comenges et al., 2010, Zaldívar Comenges et al., 2012, Zaldívar Comenges et al., 2016, Zaldívar Comenges and Baraibar, 2011). The model also features a growth model, which takes into account the cell growth phases (G1, S, G2, M phases). Other features taken into account are the partitioning of a compound within the cell and the toxicity to the cell. The latter part of the model is based on the prediction of two parameters, the no-effect concentration (NEC) and the killing rate (k_r_), which are linked to experimental cell viability (Zaldívar Comenges et al., 2010, Zaldívar Comenges et al., 2012, Zaldívar Comenges et al., 2016, Zaldívar Comenges and Baraibar, 2011). The main property simulated is the real, intracellular, concentration of a specific chemical affecting the cell. Examples of simulation for different chemicals and specific cell lines can be found in Kramer (2010), Broeders et al. (2015), and Armitage et al. (2014).

On the other end we have the PBK/D model, which combines kinetic and dynamic interactions of a specific compound after oral, inhalation, or dermal exposure by applying a set of differential equations. To be more specific, PBK models describe the body as a set of interconnected compartments, which represent the human organs and blood circulation, describing the absorption, distribution, metabolism, and excretion (ADME) (Krishnan and Andersen, 2001). PBD models additionally include a description of the interaction of the chemical or its reactive metabolite with the receptor mediating the adverse effect.

In order to illustrate the integration of the VCBA outputs with a PBK model we chose the alkenylbenzene class of chemicals, including methyleugenol, safrole, and estragole, for which several PBK/D models have been built for both rodents and humans (Punt et al., 2008, Punt et al., 2009, Paini et al., 2010, Martati et al., 2011, Al-Subeihi et al., 2011, Punt et al., 2016).

**Fig. 1:**
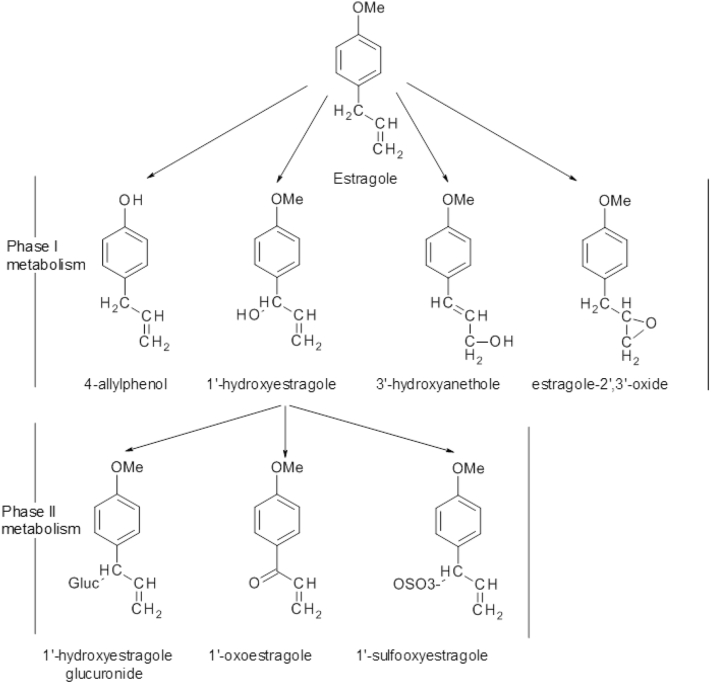
Estragole metabolic pathway in rodents and humans.

Estragole, which was selected as the gold compound for this exercise, is found in herbs and spices, to which humans are exposed at low doses via the diet. Once absorbed estragole can undergo detoxification and bioactivation through phase I and II enzymatic pathways resulting in the compound being either excreted or converted to a reactive carbocation which binds covalently to DNA. Estragole is known to produce tumours in rodents exposed to high dose levels (Miller et al., 1983) and it has been shown to be genotoxic and carcinogenic when tested in isolated form at high doses in animal experiments. The main metabolic pathway for estragole is represented in Fig. 1. The main pathway for bioactivation of estragole proceeds by initial hydroxylation on the allyl side chain by cytochrome P450 enzymes resulting in the formation of 1′-hydroxyestragole, the proximate carcinogenic metabolite. 1′-Hydroxyestragole can be detoxified by glucuronidation or via oxidation (Phillips et al., 1981, Iyer et al., 2003), the latter followed by glutathione conjugation. Alternatively, sulfonation of 1′-hydroxy estragole by sulfotransferases gives rise to the unstable metabolite 1′-sulfooxyestragole which decomposes to the reactive carbocation that covalently binds to DNA, RNA and protein (Phillips et al., 1981, Gardner et al., 1996). Several adducts are formed during the reaction of 1′-sulfooxyestragole with the guanine base in DNA including N 2-(*trans*-isoestragol-3′-yl)-2′-deoxyguanosine, N 2-(estragole-1′-yl)-2′-deoxyguanosine, 7-(*trans*-isoestragol-3′-yl)-2′-guanine and 8-(*trans*-isoestragol-3′-yl)-2′-deoxyguanosine (Phillips et al., 1981, Punt et al., 2007). The major guanine adduct formed is N 2-(*trans*-isoestragol-3′-yl)-2′-deoxyguanosine (E-3′-N 2-dGuo) which is considered to play a role in the genotoxic and carcinogenic effects induced by estragole (Smith et al., 2002, Phillips et al., 1981).

**Fig. 2:**
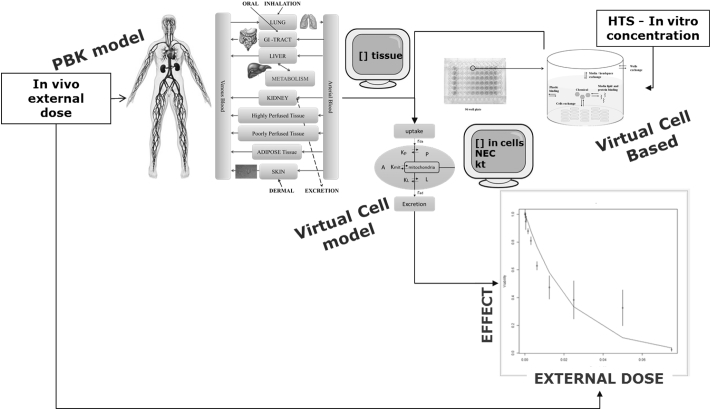
Schematic overview of the link between the VCBA and a PBK model and the possibility to extrapolate from an in vitro concentration to an in vivo external dose, and from an external dose to internal response. Light grey arrow represents forward extrapolation (external dose to internal effect), black arrow reverse extrapolation (internal effect to external dose).

In the present work, we illustrate how by linking the VCBA to the PBK model developed for estragole (Punt et al., 2009, Punt et al., 2016) we can achieve a new refined PBK/D model that can be used for in vitro to in vivo extrapolation of endpoints, cell viability and DNA adducts; a schematic representation of the approach is given in Fig. 2. The PBK/D model was implemented in KNIME to achieve an open source automated ready to use tool for extrapolation.

## 2 Materials and methods

Estragole (Cas. 140-67-0) and DMSO were purchased from Sigma. TMRE, Toto 3, and Hoechst 33342 for cellomic analysis were purchased from Invitrogen. Cryo preserved Human Cell Line HepaRG was obtained from INSERM's laboratory U522 and a cell culture bank had been established in-house at the Joint Research Centre. The concentration range of estragole was 0–75 mM starting from a 100 mM stock solution in cell exposure medium.

### 2.1 Viability and mitochondrial membrane potential in HepaRG

For the purpose of the current study, an in-house experiment using HepaRG exposed to different concentrations of the selected chemicals for 24 h was carried out. Briefly, HepaRG cells were seeded at a density of 2.6 × 10^4^ cells/cm^2^ in a growth medium composed of Williams E medium supplemented with 10% FCS, 100 units/mL penicillin, 100 μg/mL streptomycin, 5 μg/mL insulin, 2 mM glutamine and 5 × 10^− 5^ M hydrocortisone hemisuccinate. Further culturing was carried out for 2 more weeks with the same medium supplemented with 2% DMSO in 75 cm2 culture flask. The medium was renewed every 2 to 3 days. After differentiation, HepaRG cells were detached by gentle trypsinization, and then seeded at a density of 4–5 × 10^4^/well in 96 well microtiter plates (BD Biosciences) to allow the selection of hepatocyte-like populations. The cells were used for testing within one week after plate seeding. Compounds were diluted in culture medium with 5% HyClone Fetalclone III serum to obtain a final concentration of DMSO of 0.1%, 100 μL of each concentration for each compound were used. After 24 h of exposure, treated HepaRG cells were stained with TMRE, Toto 3, and Hoechst 33342 for 30 min. This assay was performed three times in triplicate (biological & technical replicates).

Viability and mitochondrial membrane potential (mmp) were assessed with a high-content analysis (HCA) approach using Cellomics™ ArrayScan vTi (Thermo Scientific, Pittsburgh, PA, USA). A magnification of 10 image field per well for two fluorescence channels with the XF93 filter set were collected. Cell count analysis was performed using the Target Activation Bioapplication v.4 from Cellomics™ Scan Software (Mennecozzi et al., 2011) on 10 fields taking into account around 0.8–1 × 10^4^ cells.

### 2.2 PBK model extension

To study the bioactivation and detoxification of estragole a human PBK model was built by Punt et al. (2009). We used this published human PBK model as the basis of our work. The PBK model calculates the internal concentration of estragole at organ level (CL) in the human body following a specified exposure scenario. We included the Virtual Cell (VC) model, which is the cellular part of the VCBA, into the PBK model of Punt et al. (2009) to simulate the intracellular concentration.

### 2.3 The virtual cell model

In the VC, the cell is modelled as four compartments: lipid, protein, water and mitochondria (Worth et al., submitted) where the uptake of the chemical across the cell membrane occurs by passive diffusion into the aqueous compartment followed by distribution into the three other compartments of the cell by instantaneous partitioning. When the chemical enters into the cell a toxicodynamic process occurs which is governed by two parameters: No Effect Concentration (NEC), killing rate (k_r_). These parameters are calculated prior to the PBK-VCBA simulation by optimization of predicted cell viability by the VCBA with the experimental (HTS) in vitro data. In this way the model can predict the cell viability in the liver after a given dose. The parameters of the heptatocyte (HepaRG) VC are given in Table 1. A cell growth model was also derived, however it was set to initial G1 phase, since the HepaRG cell line does not proliferate. Furthermore, the in vitro fate part of the VCBA needs some input parameters (Log P, air and water degradation, Molar Volume, MW, Henry constant, and atomic diffusion) which were computed by EPI Suite (version 4.1), however experimental values were preferred wherever available.

**Table 1:** Parameters of the HepaRG virtual cell model included in the PBK model.

Parameter type	Abbreviation used in the model	Value	Units	Ref.
Mass fraction of compartment f_x_ (aq-*aqueous*, L-lipids, P-proteins)	f_aq_	0.72	% weight	(Zaldívar Comenges et al., 2016)
	f_L_	0.012		
	f_P_	0.268		
Uptake rate	r_up_	35.208	L m^− 2^ h^− 1^	
Elimination rate	r_el_	35.208	L m^− 2^ h^− 1^	
Wet weight	W	1.79 ∙ 10^− 9^	g	
Volume of the cell	V	1.67 ∙ 10^− 15^	m^3^	
Cell cycle^a^ duration	G1	849	h	
Mortality	M	1.19 ∙ 10^− 6^	h^− 1^	


Phase I & II metabolism: (from Punt et al., 2009)		
	V_max_^b^	K_m_^c^
1′-Hydroxyestragole	1.4	21
4′Allylephenol	0.7	290
Estragole-2′,3′-oxide	1.6	83
3′-Hydroxyanethole	2.6	350
1′-Hydroxyestragole glucuronidation	0.6	708
1′-Oxoestragole	9.4	354
1′-Sulfooxyestragole	0.06	727

a HepaRG cell do not proliferate the models keeps only one cell cycle step G1.

b Scaled Vmax (app) expressed as μmol h^− 1^ (g tissue)^− 1^, calculated from the in vitro Vmax based on a microsomal protein yield of 32 mg/g tissue for liver and an S9 protein yield of 143 mg/g liver (Punt et al., 2009).

c K_m_ in μM.

The concentration of estragole reaching the liver will enter the cell. By simulation the virtual cell model takes into account the total number of moles of a compound (*n*_*tot*_) in the cell as the sum in the different compartments, water, protein, lipid, and mitochondria. The model representing the cell was added to the PBK model, and is described by the following equations:

(1) $$n_{\mathrm{tot}}=n_{\mathrm{aq}}+n_{\mathrm{PC}}+n_{\mathrm{LC}}+n_{\mathrm{mit}}=\left(V_{\mathrm{aq}}.C_{\mathrm{aq}}+V_{\mathrm{PSC}}.C_{\mathrm{PC}}+V_{\mathrm{LC}}.C_{\mathrm{LC}}+V_{\mathrm{mit}}C_{\mathrm{mit}}\right)$$

where the V_i_'s refer to the compartment volumes (l) and the C_i_'s refer to the concentration in the compartments (mol·l^− 1^), water, protein, lipid and mitochondria. Also the total number of moles of a chemical can be expressed as:

(2) $$n_{\mathrm{tot}}=W.C_{b}/\mathrm{MW}$$

where W is the cell weight (g), MW is the molecular weight of the chemical (g·mol^− 1^) and C_b_ is the chemical concentration in the cell (g·gw·w^− 1^).

The chemical is assumed to be in equilibrium between the different compartments with fixed values partition coefficients: $K_{\mathrm{PC}}=\frac{C_{\mathrm{PC}}/\left[\mathrm{PC}\right]}{C_{\mathrm{aq}}}$; $K_{\mathrm{LC}}=\frac{C_{\mathrm{LC}}/\left[\mathrm{LC}\right]}{C_{\mathrm{aq}}};\;K_{mit}=\frac{C_{mit}}{C_{aq}}$. For the K_mit_ see Worth et al., submitted.

The time evolution of this substance in the cell can be calculated by a simple mass balance, assuming that the uptake and elimination rates r_up_ and r_el_ (l·cm^− 2^·s^− 1^) are proportional to the surface area of the cell (passive diffusion) and the transfer occurs through the aqueous compartment only as:

(3) $$\frac{{\mathrm{dn}}_{\mathrm{tot}}}{\mathrm{dt}}=V^{2/3}\left(r_{\mathrm{up}}.C_{\mathrm{dis}}-r_{\mathrm{el}}.C_{b}\right)$$

where C_dis_ and C_aq_ refer to the chemical concentration (mol·l^− 1^) outside of the cell and in the aqueous compartment of the cell (mol·l^− 1^), respectively. Appling the chain rule of differentiation to Eq. (2) we have:

(4) $$\frac{{\mathrm{dn}}_{\mathrm{tot}}}{\mathrm{dt}}=\frac{1}{\mathrm{MW}}\left(W\frac{{\mathrm{dC}}_{b}}{\mathrm{dt}}+C_{b}\frac{\mathrm{dW}}{\mathrm{dt}}\right)$$

and rearranging terms we obtain:

(5) $$\frac{{\mathrm{dC}}_{b}}{\mathrm{dt}}=\frac{\mathrm{MW}.V^{2/3}}{W}\left(r_{\mathrm{up}}.C_{\mathrm{dis}}-r_{\mathrm{el}}.C_{\mathrm{aq}}\right)-\frac{C_{b}}{W}\frac{\mathrm{dW}}{\mathrm{dt}}$$

the last term represents the dilution due to growth of the cell that in the case of non-proliferating (Fe = 0) cells this can be neglected.

Since the concentration in the aqueous fraction C_aq_ is not a value that is measured, then we have to convert in terms of C_b_ using the partitioning approach. The wet weight, W can also be expressed as a function of the volumes of the different compartments:

(6) $$W=\rho .V=\rho \left(V_{\mathrm{aq}}+V_{\mathrm{PC}}+V_{\mathrm{LC}}+V_{\mathrm{mit}}\right)$$

On the other hand:

(7) $$V_{\mathrm{PC}}=W_{\mathrm{PC}}/\rho_{\mathrm{PC}}$$

(8) $$V_{\mathrm{LC}}=W_{\mathrm{LC}}/\rho_{\mathrm{LC}}$$

(9) $$V_{\mathrm{mit}}=W_{\mathrm{mit}}/\rho_{\mathrm{mit}}$$

(10) $$V_{\mathrm{aq}}=W_{\mathrm{aq}}/\rho_{\mathrm{aq}}$$

where W_PC,_ W_LC,_ W_mit,_ and W_aq_ are the masses of proteins, lipids and aqueous compartments in the cells and ρ_P_, ρ_L_, ρ_mit_ and ρ_aq_ their densities.

To find the relation between C_aq_ and C_b_ we have to combine n_tot_ in Eqs. (1), (2), the partition coefficients and Eqs. (7), (8), (9), (10), then we have:

(11) $$C_{\mathrm{aq}}=\frac{C_{b}}{\mathrm{MW}.\left(\frac{f_{\mathrm{aq}}}{\rho_{\mathrm{aq}}}+\frac{f_{\mathrm{LC}}}{\rho_{\mathrm{LC}}}K_{\mathrm{LC}}\left[\mathrm{LC}\right]+\frac{f_{\mathrm{PC}}}{\rho_{\mathrm{PC}}}K_{\mathrm{PC}}\left[\mathrm{PC}\right]+\frac{f_{\mathrm{mit}}}{\rho_{\mathrm{mit}}}K_{mit}\right)}$$

where f_i_ refer to the mass fraction of each compartment (aqueous, lipid, protein) in the cell. Replacing this equation into Eq. (5) and rearranging we obtain:

(12) $$\frac{{\mathrm{dC}}_{b}}{\mathrm{dt}}=\left(\frac{\mathrm{MW}.V^{2/3}}{W}r_{\mathrm{up}}\right)C_{\mathrm{dis}}-\left(\frac{V^{2/3}}{W.\left(\frac{f_{\mathrm{aq}}}{\rho_{\mathrm{aq}}}+\frac{f_{\mathrm{LC}}}{\rho_{\mathrm{LC}}}K_{\mathrm{LC}}\left[\mathrm{LC}\right]+\frac{f_{\mathrm{PC}}}{\rho_{\mathrm{PC}}}K_{\mathrm{PC}}\left[\mathrm{PC}\right]+\frac{f_{\mathrm{mit}}}{\rho_{\mathrm{mit}}}K_{mit}\right)}r_{\mathrm{up}}\right)C_{b}-\left(\frac{1}{W}\frac{\mathrm{dW}}{\mathrm{dt}}\right)C_{b}$$

However in this case uptake and elimination rates are not constant, but depend on the status of the cell and take into account the differences in growth. The variation of the wet weight, *W*, as a function of time can be obtained, assuming constant composition and hence density, as:

(13) $$\frac{\mathrm{dW}}{\mathrm{dt}}=\rho \frac{\mathrm{dV}}{\mathrm{dt}}$$

If we consider spherical shape $A=\frac{4}{3}\pi r^{3}$ and von Bertalanffy's growth curve (Zaldívar Comenges and Baraibar, 2011),

(14) $$r\left(t\right)=r_{\infty }-\left(r_{\infty }-r_{0}\right)e^{-\alpha_{G}\;t}\;$$

where *r*_0_ and *r*_∞_ refer to the initial and final cell radius and *α*_*G*_ is the von Bertalanffy's growth rate. Then we have:

(15) $$\frac{\mathrm{dV}}{\mathrm{dt}}=4\cdot \pi {\left[r_{\infty }-\left(r_{\infty }-r_{0}\right)\exp\left(-\alpha_{G}\cdot t\right)\right]}^{2}\left(r_{\infty }-r_{0}\right)\cdot \alpha_{G}\cdot \exp\left(-\alpha_{G}\cdot t\right)$$

However, the introduction of this term considers only a single cell developing during the simulation. To consider the whole population of the cells, instead of a single cell, we take average values for the weight, its derivative, and the surface depending on the four stages: G1, S, G2, M. The model in Eq. (12) has several parameters that need to be evaluated. The uptake and elimination rates, r_up_ and r_el_, and the partition coefficients, K_L_ and K_P_, depend on the compound; whereas the remaining parameters depend on the type of cell.

The metabolism occurs inside the cell and, therefore, this process is included in the chemical mass balance inside the cell. In any case, we can assume the same principle and write:

(16) $$F_{\mathrm{met}}=k_{\mathrm{met}}\cdot C_{b}$$

where *C*_*b*_ refers to the cell internal concentration (g gww^− 1^) and k_*met*_ is the metabolism rate constant (s^− 1^). However, a better expression is provided using the Michaelis-Menten equation:

(17) $$F_{\mathrm{met}}=\frac{V_{\max}\cdot C_{b}}{K_{M}+C_{b}}$$

where *V*_*max*_ (s^− 1^) represents the maximum rate achieved by the system and *K*_*M*_ is the substrate concentration at which the reaction rate is half of *V*_*max*_. Eq. (17) was used to describe phase I metabolism of estragole, the values of *V*_*max*_ and *K*_*m*_ for each path of estragole metabolism were taken from Punt et al. (2009), and values are reported in Table 1.

The complete R script, linking PBK with the VCBA can be found in the Appendix.

### 2.4 Optimization error

The optimization error was calculated using the following equation by the VCBA:

(18) $$\mathrm{error}=\sum_{i=1}^{n\;\exp}{\left({\mathrm{viability}}_{\exp}-{\mathrm{viability}}_{\mathrm{sim}}\right)}^{2}=\;\sum_{i=1}^{n\;\exp}{\left({\mathrm{viability}}_{\exp}-{\left({\mathrm{nc}}_{\mathrm{tot},t}/{\mathrm{nc}}_{\mathrm{tot},t=0}\right)}_{\mathrm{sim}\;}\right)}^{2}$$

With viability defined as the % of cells surviving at the end of the experiment. This step is performed first to obtain the NEC and K_r_ to be used in the joint PBK/D–VCBA. For more information on the optimization step, see Zaldívar Comenges et al. (2016, in press).

### 2.5 PBD model – DNA adduct formation

To calculate the DNA adducts formed from the estragole intracellular concentration, a linear equation passing through zero was used:

19 Y=A∗X

Y is the number of DNA adducts (#Adducts) formed and X is the concentration of 1′-sulfoxyestragole (nmol/g liver), A is a factor described in Paini (2012b) which allows to extrapolate from the levels of the sulfoxy derivative to the number of DNA adducts (Paini et al., 2012a), and AMHES is the concentration of 1′-sulfoxyestragole (nmol/g liver). So we can introduce the following equation for DNA adduct formation: #Adducts = 32.6 ∗ (AMHES) (Punt et al., 2016).

### 2.6 KNIME workflow

**Fig. 3:**
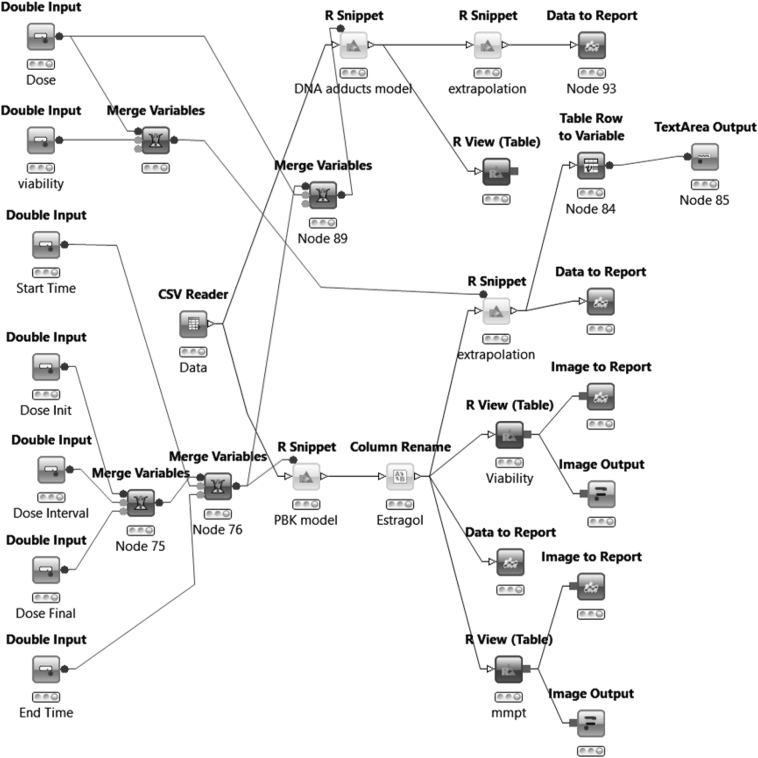
KNIME workflow to perform IVIVE, for human exposure, based on the estragole PBD model.

Development of a KNIME workflow automates the modelling thereby facilitating the application in the risk assessment of chemicals. KNIME is a user-friendly graphical workbench for the analysis process such as data access, data transformation, investigation, visualization and reporting (http://www.knime.org/). R is a language and environment for statistical computing and graphics (http://www.r-project.org/). The PBK model from Punt et al. (2009) was converted from Berkeley Madonna (www.berkeleymadonna.com) to R language in order to be compatible with the KNIME R snippet node within the workflow. The PBD model was implemented as an open source platform using KNIME and R programs (which are all freely available). The workflow is divided into an input zone (where the end user will input the values for each descriptor/parameter), a core zone (where the PBD model is stored) and output zone, where graphical representation and table to extrapolate from the simulate curve can be found (Fig. 3). This workflow is available from the COSMOS Space website (http://cosmosspace.cosmostox.eu/).

## 3 Results

To run the VCBA, to obtain the NEC, and k_r_, the EPI suite software (version 4.1) was used to achieve the parameters needed, (Table 2). Furthermore, in vitro testing to optimize the model was performed.

**Table 2:** Physicochemical parameters (EPI Suite predictions) for estragole, used as input to the VCBA.

Chem. name	Cas #	Log Kow	Molar volume(cm^3^ mol^-1^)	Atomic diffusion
Estragole	140-67-0	3.47	136.99	93.56

	MW (g mol^-1^)	Water degradation(s^-1^)	Air degradation (s^-1^)	Henry constant (Pa m^3^ mol^-1^)
	148.2	2.14E-07	4.91E-05	4.68E + 01

	NEC (g gww^-1^)	k_r_ (s^-1^)		
	0.00533	0.0469		

**Fig. 4:**
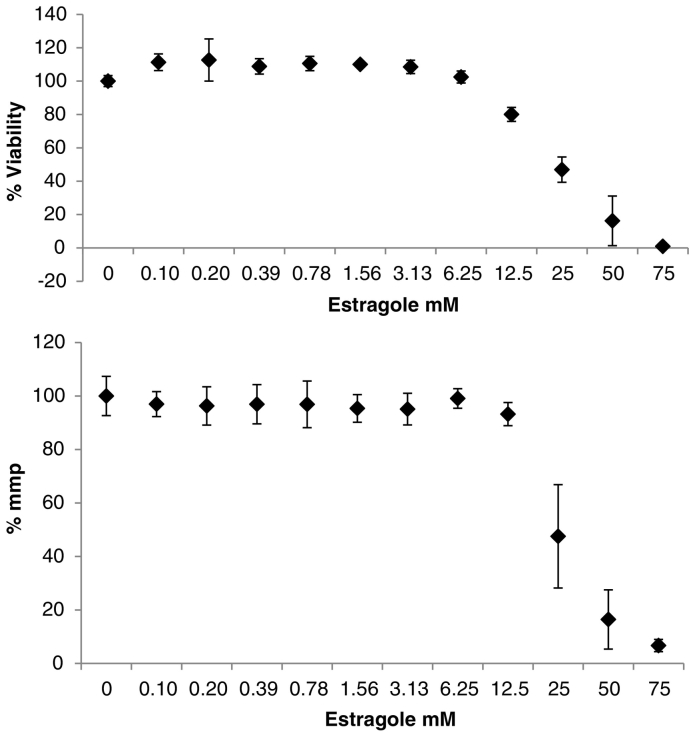
Viability (4A) and mmp (4B) in percentage results after 24 h exposure to different concentrations of estragole (mM) in HepaRG, (Y-axis I normalized to control at 0 mM, for Cell viability is represented by % of cell death and for mmp by ΔΨ).

Fig. 4A and B show the estragole concentration response for cell viability and mmp tested in HepaRG. The results are the average of five biological replicates, for each biological replicate only one measurement was possible (so 1 technical replicate). Cell viability and mitochondrial membrane potential (mmp) were measured by means of Cellomics™. After exposure of 24 h to estragole a rapid decrease was registered for both readouts. Cell viability starts to drop at 12.5 mM and mmp at 25 mM.

The Cell viability experimental results were used to optimize the NEC and k_r_ resulting in the following values 0.00533, and 0.0469, respectively (see also Table 2).

**Fig. 5:**
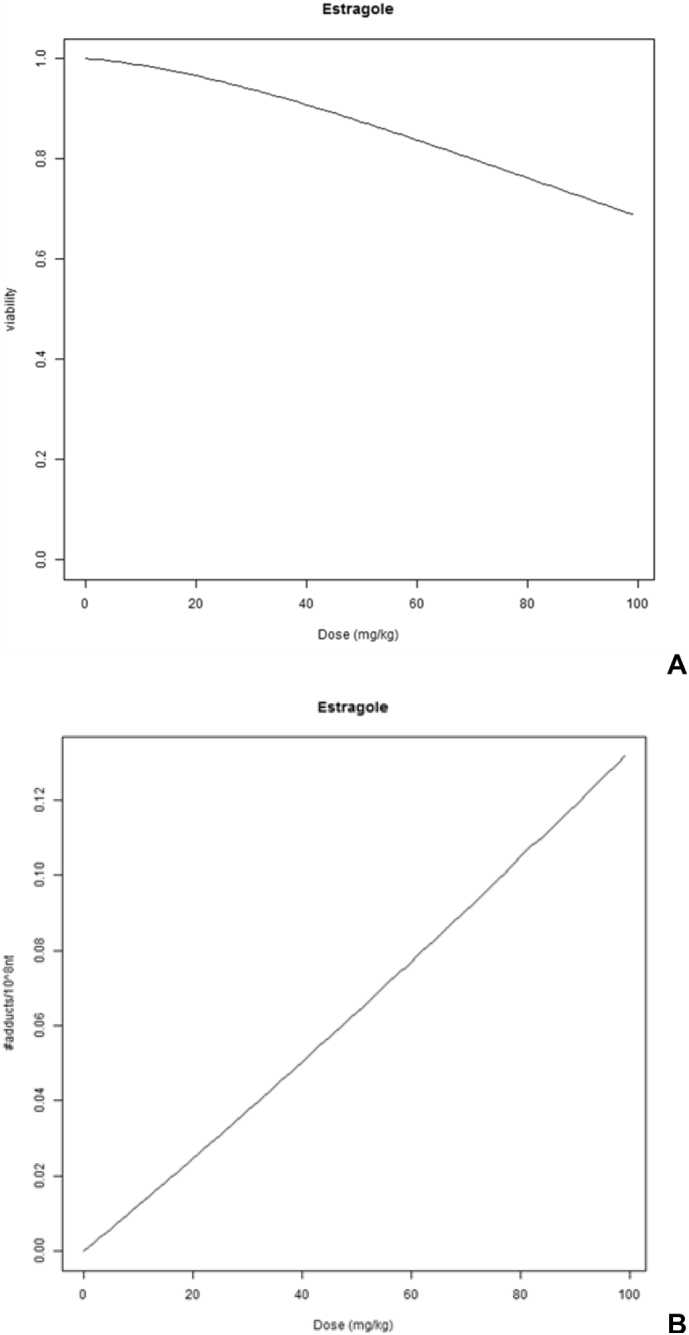
Estragole dose response predictions using the automated human estragole PBK/D model to perform IVIVE (KNIME workflow), readouts for A. Cell viability (viability is normalized to 1, with no concentration of estragole 100% of the cell as viable) and B. DNA adducts (Y-axis in adducts/10^8 nucleotide).

The KNIME workflow allows the user to process the simulation in a fast and automated way. Thus, the parameters (listed in Table 2, plus NEC and k_r_) obtained were inputted in the KNIME workflow developed for estragole, that can be divided into three parts (left, central, right). On the left side the nodes, are accessible by the user to input the dose range (e.g. 0–1000 mg/kg bw) that they wish to apply and the simulation time (e.g. 0–24 h). The central part (the core) represents the R script, the mathematical representation of the model. Finally, the right side of the workflow is built with nodes that elaborate the model outputs into a graphical representation (Fig. 5A and B). Within the PBK/D model KNIME workflow (Fig. 3) an extension was made to be able to translate the in vitro concentration response curve to an external dose response curve and by extrapolation the actual quantified effect (in vitro) to an external dose (in vivo). This is called in vitro to in vivo extrapolation (IVIVE), or reverse dosimetry. The model can be also applied in forward dosimetry going from the selected exposure dose to the internal effect. For instance for the average dietary human daily intake for estragole (0.07 mg/kg bw, EU-SCF, 2001) a viability value of 1 was found, meaning that this dose would not harm the liver cells, assuming HepaRG cells are representative of the human liver, and adducts formed are 0.00084/10^8^ nt (nucleotide, nt). However if we increase the dose to 100 mg/kg bw, a reduction in fraction of viable cells to 0.7 is observed, indicating that estragole at this dose is inducing liver cell damage, with the number of adducts increasing to 0.133/10^8^ nt.

Due to the fact that estragole is known not to induce cell damage directly (only at high concentration and/or doses) we studied the possibility to extend the human PBK/D model to simulate the metabolites of estragole, by taking the PBD model from Paini, 2012b and revised in Punt et al. (2016) and by correcting the simulation using the intracellular concentration. This was done as follows:

1. Estragole enters the liver and reaches the hepatocytes (concentration in the liver, CL).
2. Estragole enters the cells by passive diffusion and partitions between the cellular compartments. The model simulates the intracellular concentration, within the cell model we included the equation describing metabolism for phase I and II.
3. Then the starting concentration for metabolism of estragole becomes the intracellular concentration obtained with the VCBA, since metabolism is occurring in the cells. We assumed that HepaRG is a good in vitro surrogate of hepatocytes.
4. By introducing in the PBK/D model a simple linear formula, the number of DNA adducts was estimated based on the relationship between sulfonation in humans (Punt et al., 2016) and DNA adduct formation measured in vivo in rodents (Paini et al., 2012a). The DNA adduct simulation was previously done in rodents and the PBD model was validated in vivo (Paini et al., 2010, Paini, 2012b). The results showed that after exposure to estragole for 24 h a rapid decrease was simulated for cell viability (Fig. 5A). The model was also able to simulate DNA adduct formation, showing an increase with exposure dose (Fig. 5B).

## 4 Discussion

Estragole is an alkenylbenzene that can be found in spices and herbs, and due to its aniseed it was once used as a flavouring agent in confectionary. Although it is prohibited to add estragole to food products as such, due to its genotoxic properties in rodent studies (Miller et al., 1983), it is still found in the daily human diet due to its presence as a natural constituent of foods (e.g.in pesto sauce, since it is the main constituent of basil leaves). However, it can also be added to cosmetics within safe limits. Thus there is a need to develop tools to address the safety of this chemical (group). To carry out in vitro to in vivo extrapolation (IVIVE), a physiologically based kinetic/dynamic (PBK/D) model previously developed for estragole (Punt et al., 2009, Punt et al., 2016, Paini et al., 2010, Paini et al., 2012a), was selected as a case stud for integration with the virtual cell based assay (VCBA). The joint PBK/D-VCBA model describes the relationship between the tissue dose, early chemical-tissue interactions, and resulting toxic effect(s); thus it can be used to predict the toxicologically effective target organ dose.

The aim of the present study was to refine the PBK/D model to be able to i) include the mathematical description of the virtual cell in the PBK model to represent the link between in vitro and in vivo exposures; ii) link an adverse outcome (such as cytotoxicity) and; iii) simulate the number of DNA adducts based on the simulated concentration reaching the cell.

This combination of models was previously used by Gajewska et al. (2015). In the latter study, a multi-scale computational model was built linking a PBK model for caffeine with the VCBA to relate an external oral and dermal dose with the measured in vitro HepaRG cell viability. The model simulation showed that cell viability remained almost unchanged for external human doses of caffeine (5–400 mg) by both oral and dermal absorption routes, in single exposure mode only.

In the present work estragole was chosen as a well-studied compound, since in vitro data for cell viability was obtained in house using the HepaRG cell line, while DNA adducts in humans were taken from the literature (Paini, 2012b, Punt et al., 2016). We have used the HepaRG cell line, since it was known to be a good candidate for these studies to mimic real hepatocytes (Zanelli et al., 2012). HepaRG cells are terminally differentiated hepatic cells derived from a human hepatic progenitor cell line that retain many characteristics of primary human hepatocytes, including metabolic competence. The novel part was to extend the human PBK/D model from Paini, 2012b (revised by Punt et al., 2016) and correct it for the intracellular concentration. The results showed that after exposure of 24 h to estragole a rapid decrease was simulated for cell viability. The concentration at which this occurred (12.5 mM) can be regarded as the point of departure for extrapolation. At the same time the concentration of estragole that entered the cell was lower, thus, the DNA adduct formation was also lower compared to what was reported by Punt et al. (2016); as an example, for an exposure dose of 0.01 mg/kg the simulation resulted in 0.001 adducts/10^8^ nts versus 1.2 adducts/10^8^nts reported in Punt et al., showing a 2 fold lower prediction as compared to what was previously reported this could be due to the correction of the intracellular concentration.

For the specific case of estragole, we could investigate further the liver zonation in human as done previously by Paini et al. (2012a) in rodents, which is especially interesting for the DNA adduct formation. Another important piece of information given by the simulation was the amount of estragole reaching the mitochondria. So far we were able only to link the experimental output to the simulated concentration (data not shown), and not yet simulate the mitochondrial membrane potential, although this will be addressed as a further refinement in the future (Worth et al., submitted). The current work did not apply Monte Carlo modelling to simulate interindividual human variation in DNA adduct formation in the population since it was recently published in Punt et al. (2016). Another interesting study would be the integration of repair mechanisms into the PBK/D model.

Finally, we have developed a user-friendly tool implementing the newly developed PBK/D model into a KNIME workflow, published in COSMOS KNIME webportal (http://cosmosspace.cosmostox.eu/). This tool (workflow) is made freely available for end-users to automate the extrapolation from an in vitro effective concentration to the corresponding human exposure dose. The workflow could in principle be adapted to include route to route extrapolation or interspecies and intraspecies extrapolation, as well as low to high dose extrapolation.

We believe that the methodology described supports efforts to shift away from the traditional approach to risk assessment based on animal experimentation to a more scientifically robust approach based on automated in vitro testing and mathematical modelling.

## Transparency Document

Transparency document. Image 2

## Appendix A Supplementary data

Supplementary material Image 3
