# Supplementary material for: From in vitro to in vivo: Integration of the virtual cell based assay with physiologically based kinetic modelling
Source: Toxicol In Vitro. 2017 Dec;45:241–8. doi: 10.1016/j.tiv.2017.06.015 (PMC5742636; doi:10.1016/j.tiv.2017.06.015)
Supplement: Supplementary file 2 — Supplementary material [file mmc2.docx]

**Appendix model equation in R**

***Title:*** From in vitro to in vivo: Integration of the Virtual Cell Based Assay with Physiologically Based Kinetic modelling

***Authors: Alicia Paini*** ^a*^***, Jose Vicente Sala Benito*** ^a^***, Jos Bessems*** ^a^***, Andrew Worth*** ^a^

**Affiliation:**

^a^ Chemical Safety and Alternative Methods Unit, EURL ECVAM, Directorate F – Health, Consumers and Reference Materials, Joint Research Centre, European Commission, Ispra, Italy

**** Corresponding author:*** alicia.paini@ec.europa.eu

Joint Research Centre

Directorate F – Health, Consumers and Reference Materials

Chemical Safety and Alternative Methods Unit

Via E. Fermi 2749, TP 126

I-21027 Ispra (VA), Italy

tel.+39-0332-78 3986

fax +39-0332-78 9963

alicia_ivive_eq <- function(t,state,parameters){

with(c(as.list(parameters)),{

NEC=0.005324187142658;

kt=0.046865796690807;

kmit<-7.28e-7

if (logKow <1.09) {

vals=1.31;

} else if (logKow>=1.09&&logKow<=4.6) {

vals=0.57*logKow+0.69;

} else if (logKow>4.6) {

vals=logKow-1.3;}

KP<-10^(vals-1.178);

# lipids partitioning

KL<-10^(1.25*logKow-3.70);

#end partirioning

logp<--1.1711+0.98*logKow-0.0011*MWcomp;

Per<-10.^logp;

rda<-Per;

rad<-rda;

#x0<-c(AS=0,AR=0,AF=0,AGI=AGI,AL=0,ALHE=0,AK=0,ALu=0,AA=0,AV=0,AUCV=0,AUCLHE=0,cb=cb,n=n);

AS<-state[1]

AR<-state[2]

AF<-state[3]

AGI<-state[4]

AL<-state[5]

ALHE<-state[6]

AK<-state[7]

ALu<-state[8]

AA<-state[9]

AV<-state[10]

AUCV<-state[11]

AUCLHE<-state[12]

cb<-state[13]

n<-state[14]

cbm<-state[15]

VK <- VKC*BW; # fraction of venous blood: 0.079*3/4

VRC <- 0.08-VLC-VLUC-VKC; # fraction of richly perfused tissue

VSC <- 0.836-VFC-VAC-VVC; # Fraction of blood flow to slowly perfused tissue

#% total of fractions = 0.916

VL <- VLC*BW; #%{L or Kg}

VLu <- VLUC*BW;

VF <- VFC*BW;

VR <- VRC*BW;

VS <- VSC*BW;

VA <- VAC*BW;

VV <- VVC*BW;

QCC <- 15; #% Info: QC=15*BW^0.74 Reference: Brown

QC <- QCC*BW^0.74; #% {L/hr}; % Info: QC=15*BW^0.74 Reference: Brown

QRC <- 0.70-QLC-QKC; #% Fraction of blood flow to richly perfused tissue

QSC <- 0.30-QFC; #% Fraction of blood flow to slowly perfused tissue

#% total of fractions = 1

QL <- QLC*QC;#% {L/hr}

QK <- QKC*QC;#%{L/hr}

QF <- QFC*QC;#% {L/hr}

QR <- QRC*QC;#% {L/hr}

QS <- QSC*QC;#% {L/hr}

L <- VLC*1000

VMaxLHE <- VMaxLHEc/1000*60*MPL*L*BW;

VMaxLAP <- VMaxLAPc/1000*60*MPL*L*BW;

VMaxLEE <- VMaxLEEc/1000*60*MPL*L*BW;

VMaxLHA <- VMaxLHAc/1000*60*MPL*L*BW;

VMaxLHEG <- VMaxLHEGc/1000*60*MPL*L*BW;

VMaxLOE <- VMaxLOEc/1000*60*S9PL*L*BW;

VMaxLHES <- VMaxLHESc/1000*60*S9PL*L*BW;

#slowly perfused tissue compartment

CA <- AA/VA;

CL <- AL/VL;

CVL <- CL/PLE;

CV <- AV/VV;

#AS = Amount estragole in slowly perfused tissue, umol

CS <- AS/VS;

CVS <- CS/PSE;

dAS <- QS*(CA-CVS);

#--------

#richly perfused tissue compartment

#AR = Amount estragole in richly perfused tissue, umol

CR <- AR/VR;

CVR <- CR/PRE;

dAR <- QR*(CA-CVR);

#----------------------

#fat compartment

#AF = Amount estragole in fat tissue, umol

CF <- AF/VF;

CVF <- CF/PFE;

dAF <- QF*(CA-CVF);

#-----------------

#uptake estragole from GI tract

#AGI = Amount estragole remaining in GI tract (umol)

dAGI <--Ka*AGI;

#----------------

#liver compartment

#estragole

#AL = Amount Estragole in liver tissue, umol

MdAMLHE <- VMaxLHE*cb/(KmLHE + cb);

MdAMLAP <- VMaxLAP*cb/(KmLAP + cb);

MdAMLEE <- VMaxLEE*cb/(KmLEE + cb);

MdAMLHA <- VMaxLHA*cb/(KmLHA + cb);

dAL <- QL*(CA-CVL)+Ka*AGI-MdAMLHE-MdAMLAP-MdAMLEE-MdAMLHA;

#1'-hydroxyestragole

#ALHE = amount 1'-hydroxyestragole in liver tissue, umol

CLHE <- ALHE/VL;

CVLHE <- CLHE/PLHE;

MdAMLHEG <- VMaxLHEG*CVLHE/(KmLHEG + CVLHE);

MdAMLHES <- VMaxLHES*CVLHE/(KmLHES + CVLHE);

MdAMLOE <- VMaxLOE*CVLHE/(KmLOE + CVLHE);

dALHE <- MdAMLHE - MdAMLHEG - MdAMLHES - MdAMLOE;

# MdAMLHE=VMaxLHE*cb/(KmLHE + cb);

# MdAMLHEG <- VMaxLHEG*CVLHE/(KmLHEG + CVLHE);

# MdAMLOE <- VMaxLOE*CVLHE/(KmLOE + CVLHE);

# MdAMLHES = MdAMLHE - MdAMLHEG - MdAMLOE;

dAUCLHE <- CLHE;

#---------------

#kidney compartment

#AK = amount estragole in liver tissue, umol

CK <- AK/VK;

CVK <- CK/PKE;

dAK <- QK*(CA-CVK);

#--------------

#lung compartment

#ALu = amount estragole in lung tissue, umol

CLu <- ALu/VLu;

CALu <- CLu/PLuE;

dALu <- QC*(CV-CALu);

#---------------------

# arterial blood compartment

#CA = Concentration arterial blood estragole

dAA <- QC*(CALu-CA);

#---------------% venous blood compartment

#CV = Concentration venous blood estragole (umol/L)

dAV <- (QF*CVF + QR*CVR + QS*CVS + QL*CVL + QK*CVK - QC*CV);

dAUCV <- CV;

f1<-(rad*Va^(2/3))/(W*((faq/raq)+(fll*KL/rl)+(fp*KP/rp)));

f2<-(MWcomp*rda*Va^(2/3))/W;

dcb<-f2*CL*MWcomp*(1e-5)-f1*cb;

caq=cb/(MWcomp*(faq/raq+(fll*KL/rl)+fp*KP/rp));# conc fase acuosa

#dcb<-2042.955*148.2*1e-6*CL-1994.588*cb

dcbm<-kmit*(caq-cbm)

if((cb-NEC)>0) {

valmax=(cb-NEC);

} else {

valmax<-0;

}

val<-kt*valmax;

dn<--val*n;

return(list(c(dAS,dAR,dAF,dAGI,dAL,dALHE,dAK,dALu,dAA,dAV,dAUCV,dAUCLHE,dcb,dn,dcbm)))})}

#url1<-substr(knime.flow.in[["url1"]],7,nchar(knime.flow.in[["url1"]]))

require(deSolve)

#Pam <- function (xcsv,cols){

# Parameters <- read.csv(file=xcsv,sep=";",row.names=1,header=TRUE)

# Pam <- Parameters[,cols]

# names(Pam) <- as.character(rownames(Parameters))

# return(Pam)}

url1<-c(BW=60,

VLC=0.026,

VKC=0.004,

VLUC=0.008,

VFC=0.214,

VAC=0.02,

VVC=0.059,

QCC=15,

QLC=0.227,

QKC=0.175,

QFC=0.052,

PLE=6.5,

PLuE=6.5,

PKE=6.5,

PFE=105,

PRE=6.5,

PSE=4.1,

PLHE=1.6,

Ka=1,

S9PL=143,

MPL=32,

VMaxLHEc=0.73,

VMaxLAPc=0.38,

VMaxLEEc=0.85,

VMaxLHAc=1.35,

KmLHE=21,

KmLAP=290,

KmLEE=83,

KmLHA=350,

VMaxLHEGc=0.3,

VMaxLOEc=4.9,

VMaxLHESc=0.007,

KmLHEG=708,

KmLOE=354,

KmLHES=727,

MWE=148.2,

MWHE=164.2,

Va=1.67E-15,

W=1.19E-09,

faq=0.72,

raq=1000,

fll=0.012,

rl=900,

fp=0.268,

rp=1350,

MWcomp=148.2,

logKow=3.47,

Starttime=0,

Stoptime=24)

parameters<-url1

BW=60

VLC=0.026

VKC=0.004

VLUC=0.008

VFC=0.214

VAC=0.02

VVC=0.059

QCC=15

QLC=0.227

QKC=0.175

QFC=0.052

PLE=6.5

PLuE=6.5

PKE=6.5

PFE=105

PRE=6.5

PSE=4.1

PLHE=1.6

Ka=1

S9PL=143

MPL=32

VMaxLHEc=0.73

VMaxLAPc=0.38

VMaxLEEc=0.85

VMaxLHAc=1.35

KmLHE=21

KmLAP=290

KmLEE=83

KmLHA=350

VMaxLHEGc=0.3

VMaxLOEc=4.9

VMaxLHESc=0.007

KmLHEG=708

KmLOE=354

KmLHES=727

MWE=148.2

MWHE=164.2

Va=1.67E-15

W=1.19E-09

faq=0.72

raq=1000

fll=0.012

rl=900

fp=0.268

rp=1350

MWcomp=148.2

logKow=3.47

Starttime=0

Stoptime=24

R=8.3144621;# J/K.mol

T=293; #K

FF=9.64853399e4; #C mol-1.

logKow<-parameters["logKow"]

MWcomp<-parameters["MWcomp"]

Va<-parameters["Va"]

W<-parameters["W"]

faq<-parameters["faq"]

raq<-parameters["raq"]

fll<-parameters["fll"]

rl<-parameters["rl"]

fp<-parameters["fp"]

rp<-parameters["rp"]

MWE<-parameters["MWE"]

BW<-parameters["BW"]

QC<-parameters["QC"]

#parameters <- c(Pam(xcsv =url1,cols=1),f1=f1,f2=f2)

#Starttime<-$${DtimeStart}$$

#Stoptime<-$${DtimeEnd}$$

t<-seq(from=knime.flow.in[["timeStart"]],to=knime.flow.in[["timeEnd"]]);

GDOSE<-seq(from=knime.flow.in[["doseInit"]],to=knime.flow.in[["finalDose"]],by=knime.flow.in[["interDose"]])

#resAL<-zeros(length(GDOSE),3);

resAL<-array(0,dim=c(length(GDOSE),5))

#alfa<-14.964

alfa<-5.6

fil<-length(GDOSE)

##############################

VK <- VKC*BW; # fraction of venous blood: 0.079*3/4

VRC <- 0.08-VLC-VLUC-VKC; # fraction of richly perfused tissue

VSC <- 0.836-VFC-VAC-VVC; # Fraction of blood flow to slowly perfused tissue

#% total of fractions = 0.916

VL <- VLC*BW; #%{L or Kg}

VLu <- VLUC*BW;

VF <- VFC*BW;

VR <- VRC*BW;

VS <- VSC*BW;

VA <- VAC*BW;

VV <- VVC*BW;

QCC <- 15; #% Info: QC=15*BW^0.74 Reference: Brown

QC <- QCC*BW^0.74; #% {L/hr}; % Info: QC=15*BW^0.74 Reference: Brown

QRC <- 0.70-QLC-QKC; #% Fraction of blood flow to richly perfused tissue

QSC <- 0.30-QFC; #% Fraction of blood flow to slowly perfused tissue

#% total of fractions = 1

QL <- QLC*QC;#% {L/hr}

QK <- QKC*QC;#%{L/hr}

QF <- QFC*QC;#% {L/hr}

QR <- QRC*QC;#% {L/hr}

QS <- QSC*QC;#% {L/hr}

L <- VLC*1000

VK <- VKC*BW; # fraction of venous blood: 0.079*3/4

VRC <- 0.08-VLC-VLUC-VKC; # fraction of richly perfused tissue

VSC <- 0.836-VFC-VAC-VVC; # Fraction of blood flow to slowly perfused tissue

#% total of fractions = 0.916

VL <- VLC*BW; #%{L or Kg}

VLu <- VLUC*BW;

VF <- VFC*BW;

VR <- VRC*BW;

VS <- VSC*BW;

VA <- VAC*BW;

VV <- VVC*BW;

QCC <- 15; #% Info: QC=15*BW^0.74 Reference: Brown

QC <- QCC*BW^0.74; #% {L/hr}; % Info: QC=15*BW^0.74 Reference: Brown

QRC <- 0.70-QLC-QKC; #% Fraction of blood flow to richly perfused tissue

QSC <- 0.30-QFC; #% Fraction of blood flow to slowly perfused tissue

#% total of fractions = 1

QL <- QLC*QC;#% {L/hr}

QK <- QKC*QC;#%{L/hr}

QF <- QFC*QC;#% {L/hr}

QR <- QRC*QC;#% {L/hr}

QS <- QSC*QC;#% {L/hr}

L <- VLC*1000

VMaxLHEc=0.73

VMaxLAPc=0.38

VMaxLEEc=0.85

VMaxLHAc=1.35

KmLHE=21

KmLAP=290

KmLEE=83

KmLHA=350

VMaxLHEGc=0.3

VMaxLOEc=4.9

VMaxLHESc=0.007

KmLHEG=708

KmLOE=354

KmLHES=727

MWE=148.2

MWHE=164.2

VMaxLHE <- VMaxLHEc/1000*60*MPL*L*BW;

VMaxLAP <- VMaxLAPc/1000*60*MPL*L*BW;

VMaxLEE <- VMaxLEEc/1000*60*MPL*L*BW;

VMaxLHA <- VMaxLHAc/1000*60*MPL*L*BW;

VMaxLHEG <- VMaxLHEGc/1000*60*MPL*L*BW;

VMaxLOE <- VMaxLOEc/1000*60*S9PL*L*BW;

VMaxLHES <- VMaxLHESc/1000*60*S9PL*L*BW;

######################################################

hesDNA<-array(0,dim=c(length(GDOSE)))

for (i in (1:length(GDOSE))) {

ODOSE <- GDOSE[i]/MWcomp*1e6

# {umol/ kg bw} #ODOSE = given dose recalculated to umol/kg bw

DOSE<-ODOSE*60*1e-3

AGI<-DOSE;

x0<-c(AS=0.0,AR=0.0,AF=0.0,AGI=AGI,AL=0.0,ALHE=0.0,AK=0,ALu=0.0,AA=0,AV=0.0,AUCV=0.0,AUCLHE=0.0,cb=0.0,n=34500*10^6,cbm=0.0);

out1 <- ode(y=x0,times=t,func=alicia_ivive_eq,parms=parameters,method="radau",atol=1e-4,rtol=1e-4)

tam<-nrow(out1)

CLHE<-out1[tam,7]/VL

CVLHE<-CLHE/PLHE

MdAMLHE<-VMaxLHE*out1[tam,14]/(KmLHE + out1[tam,14]);

MdAMLHEG <- VMaxLHEG*CVLHE/(KmLHEG + CVLHE);

MdAMLOE <- VMaxLOE*CVLHE/(KmLOE + CVLHE);

hesDNA[i] <- MdAMLHE - MdAMLHEG - MdAMLOE;

#[t1,x1]=ode45('alicia_ivive_eq',t,x0);

#resAL(i,1)=GDOSE(i);

#resAL(i,2)=x1(length(t),14);

#resAL(i,3)=x1(length(t),13);

resAL[i,1]<-GDOSE[i]

resAL[i,2]<-out1[length(t),15] #num cells

resAL[i,3]<-out1[length(t),14] #internal cell conc.

resAL[i,4]<-out1[length(t),16] #internal mitrocondria cobc.

fv<-resAL[i,2]/x0["n"]

resAL[i,5]<-alfa*fv*(R*T/FF)*log(resAL[i,3]/resAL[i,4]) #membrane potential

}

dnaADUC<-630*hesDNA

# dnaADUC<-32.6*hesDNA

knime.out<-data.frame(GDOSE,dnaADUC)
